# Dépistage néonatal du déficit en glucose 6 phosphate déshydrogénase (G6PD) en Mauritanie

**DOI:** 10.11604/pamj.2018.30.224.13947

**Published:** 2018-07-25

**Authors:** Ghaber Sidi Mohamed, Salem Mohamed Lemine, Shagh Cheibetta, Aminetou Mohamed

**Affiliations:** 1 Maurilab, Nouakchott, Mauritanie; 2 Hôpital National de Nouakchott, Mauritanie; 3 Université de Nouakchott, Mauritanie

**Keywords:** Déficit en G6PD, dépistage néonatal, consanguinité, Mauritanie, G6PD deficiency, neonatal screening, consanguinity, Mauritania

## Abstract

**Introduction:**

Une étude transversale et descriptive qui vise l'identification des facteurs de risques associés au déficit en G6PD, sa fréquence et sa distribution géographique à Nouakchott dans l'objectif de fournir des informations utiles pour le contrôler. Comme cette maladie n'a jamais été étudiée auparavant en Mauritanie, nous avons cherché à définir le profil épidémiologique et la charge de morbidité du déficit en G6PD chez une population de nouveaux nés dans deux structures de santé de la ville de Nouakchott.

**Méthodes:**

Cette étude a été conduite dans deux hôpitaux de Nouakchott, le Centre Hospitalier Mère-Enfant et le Centre de Santé de Sebkha entre (Août - Octobre, 2015). Elle a concerné un échantillon de 523 nouveau-nés de sexes différents. Le dépistage a été fait à laide de BinaxNow G6PD Test, suivi d'une confirmation quantitative pour les cas positifs. L'analyse statistique a été réalisée par SPSS20.

**Résultats:**

Le déficit en G6PD était plus élevé chez les nouveau-nés de sexe masculin (15% vs 7% P = 0,007) et particulièrement chez les enfants d'ethnie noire (15% vs 8 % P = 0,001). La prévalence du phénotype déficient en G6PD dans la population étudiée est 11,09% (58/523).

**Conclusion:**

La présente étude est, à notre connaissance, la première de son genre sur le déficit en G6PD chez la population mauritanienne. Elle fournit des informations importantes sur le caractère épidémiologique du déficit en G6PD dans la région de Nouakchott. Elle relève une variabilité dans l'apparition de G6PD au niveau des groupes ethniques.

## Abstract (en)

**Introduction:**

We conducted a cross-sectional descriptive study aiming to identify risk factors associated with G6PD deficiency, its frequency and geographic distribution in Nouakchott, in order to provide useful informations to monitor it. As this disease has never previously been studied in Mauritania, we tried to define the epidemiological profile and the burden of morbidity related to G6PD deficiency in a newborn population in two health structures in the city of Nouakchott.

**Methods:**

This study was conducted in two hospitals in Nouakchott, at the Maternity and Infant Hospital and at the Health Center in Sebkha between August and October 2015. A sampling of 523 newborns having different sexes was enrolled in the study. Screening was based on BinaxNow G6PD test, followed by quantitative confirmation in positive patients. Statistical analysis was performed using SPSS20.

**Results:**

G6PD deficiency was higher in male newborns (15% vs 7% p = 0.007) and, in particular, in black children (15% vs 8% p = 0.001). The prevalence of G6PD deficiency in the study population was 11.09% (58/523).

**Conclusion:**

To our knowledge, this is the first study on G6PD deficiency in the Mauritanian population. It provides important informations on the epidemiological features of G6PD deficiency in the region of Nouakchott. A degree of variability exists in the occurrence of G6PD deficiency in the ethnic groups.

## Introduction

La Glucose-6-phosphate déshydrogénase (G6PD) est une enzyme hautement polymorphe codée par un gène lié au chromosome X (Xq2.8) [1]. Cette enzyme catalyse la première réaction de la voie des pentoses phosphates pour convertir le glucose-6-phosphate en 6-phosphogluconate avec la production de NADPH. Cette réaction se passe surtout dans les globules rouges. Elle favorise la détoxification des radicaux libres, protège la cellule contre les dommages induits par le peroxyde d'hydrogène et assure un profil d'équilibre oxydant au sein de la cellule [2]. Les érythrocytes ont une prédisposition à des environnements oxydés en raison de leur manque de mitochondries, offrant à la G6PD un rôle majeur dans leur stabilité [3]. Le déficit en G6PD est la déficience enzymatique la plus fréquente chez l'homme. Elle affecte environ 400 millions de personnes dans le monde entier. La migration humaine pourrait expliquer la présence de G6PD avec des fréquences variables dans les populations à travers le monde [1, 4-6].

Le déficit en G6PD est très répandu en Afrique, au Moyen-Orient, en Méditerranée et en Asie du Sud [7]. Parmi 200 variantes de G6PD décrites dans le monde, la variante G6PD A- (G202A/A376G) prédomine en Afrique sub-saharienne où elle affecte 15 à 20% de la population africaine [2]. Des études moléculaires ont montré que les carences en G6PD sont provoquées presque toujours par des substitutions uniques d'acides aminés. De surcroît, il est très important de souligner que certaines de ces variantes, ne présentant pas une hémolyse, peuvent être asymptomatiques chez des sujets adultes (hommes et femmes). Lesquels sont souvent découverts par des moyens de dépistage de routine et dans les grands échantillons de populations réunies [8]. En outre, la génération de NADPH et la réduction du glutathion sont variablement altérées chez ces sujets, en fonction du type de mutation G6PD. Les mutations associées à une hémolyse chronique ont tendance à se regrouper du côté du domaine liaison G6PD-NADP, tandis que celles liées à une hémolyse aiguë intermittente (acute intermittent hemolysis or no hemolysis) ou non hémolyse sont dispersées dans la partie restante du gène [8, 9].

En dépit de ses inconvénients, la déficience enzymatique apporte aux personnes touchées un certain degré de protection contre le paludisme, suivant un mécanisme non encore élucidé. Certains auteurs postulent que ce mécanisme implique une sensibilité accrue à la phagocytose des érythrocytes infectés par Plasmodium en raison des niveaux élevés de stress oxydatif [10]. Pour cette raison, G6PD est plus répandue dans les régions endémiques avec le paludisme et les zones où la consanguinité est élevée compte tenu de ses motifs héréditaires [10]. Les principaux signes cliniques de déficit en G6PD sont l'anémie hémolytique aiguë et l'ictère provoqués par l'ingestion de médicaments oxydants et/ou de fèves. Suite à cela, les globules rouges éclatent entrainant une anémie brutale et sévère mettant en jeu le pronostic vital [11]. L'immaturité fonctionnelle du foie chez le nouveau-né est associée au risque d'ictère nucléaire pouvant entraîner des séquelles neurosensorielles graves et exposer au risque de décès [11]. En néonatologie, le diagnostic repose sur la positivité d'un spot test colorimétrique et doit être confirmé par la mise en évidence d'un déficit enzymatique en G6PD par dosage spectrophotométrique [12]. Le diagnostic précoce des enfants atteints permet une prise en charge efficace de l'ictère néonatal et renseigne sur les précautions à prendre pour éviter les crises hémolytiques aigues [2].

## Méthodes

### Design d'étude

Il s'agit d'une étude transversale « un instant donné », descriptive et multicentrique sur les enfants (nouveau-nés) dans deux centres de Santé à Nouakchott, le Centre Hospitalier Mère-Enfant et le Centre de Santé de Sebkha. Les deux établissements sont les centres de référence pour la santé de la reproduction et reçoivent des patients venant de toutes les régions du pays.

### Population d'étude

Critères d'inclusion: notre cohorte inclut tous les enfants nés dans les deux centres au moment de l'étude. Un consentement écrit a été obtenu auprès des parents pour le prélèvement. Critères d'exclusion: les nouveau-nés dont les prélèvements sont de qualité médiocres ont été exclus de l'étude. Le recrutement a débuté du 1^er^ Août et achevé le 31 Octobre 2015. Pendant cette période de 3 mois, nous avons recueilli: les paramètres démographiques: sexe et poids de l'enfant, région d'origine, ethnie et la consanguinité; les paramètres épidémiologiques: antécédents de déficit en G6PD, d'hémoglobinopathies dans la famille et l'ictère néonatal; les paramètres hématologiques: un prélèvement du sang du cordon, juste après l'accouchement sur un tube EDTA, a été effectué pour chaque nouveau-né. Les échantillons ont été, ensuite, traités, au plus tard 2 heures après le prélèvement, pour un dépistage du déficit en G6PD à l'aide du BinaxNow G6PD Test. La confirmation par dosage quantitatif a été réalisée pour chaque cas positif; la prévalence de G6PDd chez les enfants de sexe masculin et ceux de sexe féminin a été analysée séparément.

## Résultats

Les résultats de l'étude sont regroupés dans le Tableau 1. Ils montrent que la prévalence du phénotype déficient en G6PD dans la population étudiée est relativement supérieure à 11,09% (58/523). Le déficit en G6PD trouvé est de 15% (40/269) chez les enfants de sexe masculin et 7% (18/254) chez les enfants de sexe féminin avec une différence de (P = 0,007). La recherche des antécédents familiaux d'enzymopathies et d'hémoglobinopathies dévoile l'existence d'un seul cas de parent drépanocytaire. Une variabilité dans l'apparition de G6PD est relevée au niveau des groupes ethniques avec 15% de déficitaires en G6PD chez les enfants noirs (de différentes ethnies) contre 8% chez les enfants maures avec une différence statistiquement significative (P = 0,001).

**Tableau 1:** Les prévalences de G6PDd chez les nouveau-nés du Centre Hospitalier Mère-Enfants et le Centre de Santé de Sebkha, en Mauritanie, ainsi que le sexe et le groupe ethnique, 2013

	Déficit en G6PD		*Groupe ethnique*		*Consanguinité*	
*Sexe*	Masculin	*Féminin*	*Maures*	*Les autres ethnies (noires)*	*oui*	*non*
	40(15%)	18(7%)	25 (8%)	33(15%)	16(10%)	42(11%)
Prévalence	*P-value*	< 0.007	*P-value*	< 0.01	*P-value*	< 0.7

## Discussion

Notre enquête a révélé une prévalence du déficit en G6PD de 11,09%. Ce résultat est nettement plus faible que celui trouvé chez d’autres populations d’Afrique. Cela pourrait être expliqué par la présence de nombreux groupes ethniques en Mauritanie, c’est une mosaïque de population. En effet, les travaux sur la prévalence du déficit en G6PD en Afrique rapportent des taux de l’ordre de 31,0% au Burkina Faso [13 ], 22,5% au Congo [13], 15,7% au Mali [14] et 24,2% au Nigeria [15, 16]. A titre comparatif, les fréquences du déficit en G6PD sont de 2% en Algérie, 2.6% au Maroc et 4.4% en Tunisie [17].

Le déficit en G6PD est une affection génétique liée au sexe, car elle provient d'un gène anormal porté par le chromosome sexuel x. L’inclusion des filles dans le dépistage du déficit en G6PD est aujourd’hui recommandée dans la plupart des programmes de dépistage néonatal du défit en G6PD. Transmis par les mères, ce déficit atteint essentiellement les garçons. Les filles atteintes sont très souvent en déficit partiel. Ce constat est confirmé par nos résultats qui montrent bien que les fréquences du déficit sont largement plus élevées chez les nouveau-nés masculins 15% (40/269) que chez ceux du sexe opposé 7% (18/254) avec une différence de (P = 0,007). Ce résultat concorde avec celui trouvé chez des populations de la sous-région ouest africaine [18] et d’autres régions d’Afrique et du monde entier [19]. Une étude effectuée au Nigeria montre étonnamment une fréquence plus élevée chez les individus de sexe féminin [20].

D’autres études n’ont cependant pas trouvé de différence notable entre le déficit en G6PD chez les garçons et les filles [17]. Les auteurs de ce dernier travail émettent l’hypothèse de la sensibilité du test de dépistage utilisé qui aurait détecté de nombreuses femmes hétérozygotes ayant un phénotype normal en raison de l'inactivation aléatoire du chromosome x chez elles. Cette hypothèse est complètement exclue dans notre cas car nous avons utilisé le même test de dépistage que ces auteurs.

Au vu des données de la littérature qui précisent une nette différence entre les taux de déficit en G6PD au Maghreb par rapport au reste de l’Afrique, nous nous sommes attaqués à la marge de distribution de la maladie au sein des différentes ethnies de notre population qui se situe à cheval entre ces deux zones.

Notre étude a montré que 15% du total des enfants déficitaires en G6PD sont noirs (de différentes ethnies) contre 8% qui sont maures avec une différence statistiquement significative (P = 0,001). Cette différente de distribution de la maladie au sein de la même population, correspondante aux résultats d’autres études [19], est probablement liée à la zone d’habitation souvent de paludisme hyperendémique pour les noirs et déserte et sèche pour les maures.

En effet, la concordance parfaite entre la propagation de la maladie et celle du paludisme est largement démontrée par plusieurs auteurs. Ademowo OG et al ont démontré que la prévalence du déficit est de 5 à 25% dans les régions d’endémie de malaria (Afrique de l’Ouest 22% ; Sud et Est respectivement de 12% et de 10%) alors qu’elle est à moins de 5% dans les régions non endémiques. Il s’avère en particulier que le déficit en G6PD fragilise l’organisme vis-à-vis du plasmodium en provoquant l’hémolyse des érythrocytes infectés par le déficit à un stade précoce du développement du parasite I. Notre étude fournit des informations importantes sur le la prévalence du déficit en G6PD dans la région de Nouakchott. Elle relève une variabilité dans l'apparition de G6PD au niveau des groupes ethniques.

## Conclusion

Cette étude identifie un problème de santé publique lié à la prévalence et l'incidence de G6PD en Mauritanie. Elle place la Mauritanie parmi les pays moyennement touchés par cette pathologie. L’étude pourrait être utile pour la prévention et le contrôle du déficit en G6PD en Mauritanie. Par ailleurs, elle relève une variabilité dans l'apparition de G6PD indiquant que les noirs sont significativement plus atteints que les maures. La confirmation de cette hétérogénéité ethnique doit être précisée par des enquêtes plus larges qui tiennent compte d’autres facteurs suggérés importants en particulier le climat, la nutrition, les caractéristiques biochimiques et les hormones sexuelles. Enfin, l’étude recommande que le dépistage néonatal du déficit G6PD dans notre pays soit systématiquement intégré dans le programme national de Santé à l’image de ce qui est fait dans beaucoup d’autres pays notamment de la sous-région et du Moyen-Orient.

### Etat des connaissances actuelles sur le sujet

- Très repandu en Afrique;
- Il existe plusieurs variants de déficit en G6PD, le plus fréquent en Afrique est le variant G6PDA- (202A/376G);
- Des études ont montré que le déficit en G6PD est associé à la résistance au paludisme.

### Contribution de notre étude à la connaissance

- C'est la première étude qui fournit des informations sur le caractère épidémiologique du déficit en G6PD chez la population mauritanienne;
- Notre étude recommande le dépistage néonatal du déficit en G6PD de façon systématique en Mauritanie;
- La connaissance du statut de G6PD des patients aidera le médecin dans le choix du traitement en cas de paludisme.

## Conflits d'intérêts

Les auteurs ne déclarent aucun conflit d'intérêts.

## References

[1] Kletzien RF, Harris PK, Foellmi (1994). Glucose-6-phosphate dehydrogenase: a housekeeping enzyme subject to tissue-specific regulation by hormones, nutrients, and oxidant stress. FASEB J.

[2] Cappellini MD, Fiorelli G (2008). Glucose-6-phosphate dehydrogenase deficiency. Lancet.

[3] Allison AC (2009). Genetic control of resistance to human malaria. Curr Opin Immunol.

[4] Poggi V, Town M, Foulkes NS, Luzzatto L (1990). Identification of a single base change in a new human mutant glucose-6-phosphate dehydrogenase gene by polymerase-chain-reaction amplification of the entire coding region from genomic DNA. Biochem J.

[5] Beutler E, Vulliamy TJ (2002). Hematologically important mutations: glucose-6-phosphate dehydrogenase. Blood Cells Mol Dis.

[6] Minucci A, Moradkhani K, Hwang MJ, Zuppi C, Giardina B, Capoluongo E (2012). Glucose-6-phosphate dehydrogenase (G6PD) mutations database: review of the “old” and update of the new mutations. Blood Cells Mol Dis.

[7] Taylor SM, Fairhurst RM (2014). Malaria parasites and red cell variants: when a house is not a home. Curr Opin Hematol.

[8] Prchal JT, Gregg XT (2005). Red cell enzymes. Hematology Am Soc Hematol Educ Program.

[9] Bellamacina CR (1996). The nicotinamide dinucleotide binding motif: a comparison of nucleotide binding proteins. FASEB J.

[10] Cappadoro M, Giribaldi G, O'Brien E, Turrini F, Mannu F, Ulliers D (1998). Early phagocytosis of glucose-6-phosphate dehy- drogenase (G6PD)-deficient erythrocytes parasitized by Plasmodium falciparum may explain malaria protection in G6PD deficiency. Blood.

[11] Guellouz N, Mansour Ben I, Ouederni M, Jabnoun S, Kacem S, Mokrani, Ch, Kastally R, Chahed MK, Khrouf N (2010). Dépistage néonatal du Déficit en G6PD en Tunisie. Arch Inst Pasteur Tunis.

[12] Howes RE, Piel FB, Patil AP, Nyangiri OA, Gething PW, Dewi M (2012). G6PD Deficiency prevalence and estimates of affected populations in malaria endemic countries: a geostatistical model-based map. PLoS Med.

[13] Cappellini MD, Fiorelli G (1989). Glucose-6-phosphate dehydrogenase deficiency. Bull World Health Organ.

[14] Meissner PE, Coulibaly B, Mandi G, Mansmann U, Witte S, Schiek W (2005). Diagnosis of red cell G6PD deficiency in rural Burkina Faso: comparison of a rapid fluorescent enzyme test on filter paper with polymerase chain reaction based genotyping. Br J Haematol.

[15] Bouanga JC, Mouélé R, Préhu C, Wajcman H, Feingold J, Galactéros F (1998). Glucose-6-phosphate dehydrogenase deficiency and homozygous sickle cell disease in Congo. Hum Hered.

[16] Duflo B, Diallo A, Toure K, Soula G (1979). Glucose-6-phosphate dehydrogenase deficiency in Mali. Epidemiology and pathological aspects. Bull Soc Pathol Exot Filiales.

[17] Ademowo OG, Falusi AG (2002). Molecular epidemiology and activity of erythrocyte G6PD variants in a homogeneous Nigerian population. East Afr Med J.

[18] Monchy (2004). Déficit en G6PD, fréquence dans un groupe d’enfants d’âge préscolaire d’une région centrale du Cambodge. Med Trop (Mars).

[19] Michael Kaplan (2006). Neonatal Hyperbilirubinemia in African American Males: the importance of Glucose-6-Phosphate Dehydrogenase Deficiency. J Pediatrie.

[20] Perrine Malzac (2000). Apport du conseil génétique dans la prise en charge des pathologies constitutionnelles des globules rouges.

